# Locomotor Adaptation Deficits in Older Individuals With Cognitive Impairments: A Pilot Study

**DOI:** 10.3389/fneur.2022.800338

**Published:** 2022-05-02

**Authors:** Tana S. Pottorf, Joe R. Nocera, Steven P. Eicholtz, Trisha M. Kesar

**Affiliations:** ^1^Neuroscience Graduate Program, Emory University, Atlanta, Georgia; ^2^Department of Neurology, Emory University, Atlanta, Georgia; ^3^Department of Rehabilitation Medicine, Emory University, Atlanta, Georgia; ^4^Atlanta VA Center for Visual and Neurocognitive Rehabilitation, Atlanta, Georgia

**Keywords:** split-belt, Alzheimer's Disease, mild cognitive impairment, locomotion, walking, aging, adaptation

## Abstract

Gait dysfunction and fall risk have been well documented in people with Alzheimer's Disease (AD) and individuals with mild cognitive impairment (MCI). Normal locomotor adaptation may be an important prerequisite for normal and safe community walking function, especially in older adults with age-related neural, musculoskeletal, or cardiovascular changes and cognitive impairments. The split-belt walking task is a well-studied and robust method to evaluate locomotor adaptation (e.g., the ability to adjust stepping movements to changing environmental demands). Here, we capitalized on the split-belt adaptation task to test our hypothesis that a decreased capacity for locomotor adaptation may be an important contributing factor and indicator of increased fall risk and cognitive decline in older individuals with MCI and AD. The objectives of this study were to (1) compare locomotor adaptation capacity in MCI and AD compared to healthy older adults (HOA) during split-belt treadmill walking, and (2) evaluate associations between locomotor adaptation and cognitive impairments. Our results demonstrated a significant decrease in split-belt locomotor adaptation magnitude in older individuals with MCI and AD compared to HOA. In addition, we found significant correlations between the magnitude of early adaptation and de-adaptation vs. cognitive test scores, demonstrating that individuals with greater cognitive impairment also display a reduced capacity to adapt their walking in response to the split-belt perturbation. Our study takes an important step toward understanding mechanisms underlying locomotor dysfunction in older individuals with cognitive impairment.

## Introduction

The ability to walk without the risk of falling is a defining feature of independent community function for elderly individuals. Individuals with cognitive impairment, such as Alzheimer's Disease (AD), are reported to experience falls and loss of independence twice as often as age-matched healthy older adults (HOA) (1, 2). Many individuals who fall will experience a serious injury and have an increased likelihood of recurrent falls (3, 4). Medical costs of fall-related injuries are a large financial burden for both fall victims and the economy. In 2015 alone, medical costs for non-fatal falls reached nearly $50.0 billion and are projected to reach $100.0 billion annually by 2030 (5, 6). The average cost of hospitalization for non-fatal falls is approximately $30,000 per patient, thus causing a financial burden in addition to disrupted daily function (7). The loss of independence, risk of injury, and financial burden caused by falls necessitate an investigation of why individuals, especially those with cognitive impairments, are prone to falling.

The unimpaired nervous system enables us to ambulate in the community while smoothly navigating environmental demands such as varying terrain, obstacles, visual cues, and multi-tasking. When presented with changes or perturbations in the environment, neural circuits controlling locomotion recalibrate their output *via* sensorimotor adaptation—a process through which sensorimotor mappings update in response to errors caused by environmental perturbations or demands. Over the course of multiple exposures to such environmental perturbations, adaptation processes can aid the formation of new motor memories, contributing to flexible and robust motor behaviors (8, 9). The capacity for locomotor adaptation enables us to flexibly transition between different environments and maintain our balance in the face of perturbations, slips, and trips.

Normal locomotor adaptation may therefore be an important prerequisite for normal and safe community walking function, especially in HOA who have age-related cardiovascular or muscular deconditioning, frailty, and balance dysfunction (10). A decline in sensorimotor adaptation may explain the increased risk of falling in individuals with cognitive decline and gait disturbances. Walking is a complex motor task that integrates inter-joint and inter-limb coordination, sensory feedback, dynamic balance, and adaptation to constantly changing environmental stimuli or perturbations (11). Poor adaptation can lead to gait disturbances and subsequent increased fall risk. After-effects from the new adaptation occur if the environment reverts to the previous or baseline state, and gait must be de-adapted for disturbance-free movement (8, 9). Gait disturbances and variability have been shown to precede cognitive decline (12, 13). Individuals with mild cognitive impairment (MCI) and AD often have decreased gait speed, stride length, stride symmetry, and step regularity (14–17). However, the relationships between cognition, locomotor adaptation capacity, and gait dysfunction are poorly understood, warranting further study.

The split-belt walking task is a well-studied and robust method to evaluate locomotor adaptation, the ability to adjust stepping movements to changing environmental demands *via* trial-and-error processing. Here, we capitalized on the split-belt adaptation task to study the relationship between walking flexibility and cognitive decline. Locomotor adaptation can be systematically assessed by using a split-belt treadmill, where the speed of each leg can be controlled independently. During the split-belt adaptation task, one belt and the corresponding leg run at a different speed (e.g., twice as fast or a 2:1 speed ratio) than the other. When exposed to this 2:1 split-belt treadmill condition, the participant initially “limps” (i.e., shows inter-limb temporal and spatial asymmetry of leg motion), and within 10–15 min of split-belt walking, gait symmetry is restored (9, 18–20). The modified or recalibrated walking pattern is retained for a short period even when treadmill belt speeds are returned to normal (i.e., when the belts move at the same speed or tied-belt condition), which results in the participant limping in the opposite direction (measured as a characteristic after-effect) (8, 9, 18). In previous work, both the magnitude and rate of adaptation as well as de-adaptation (during the after-effect) provided objective measures of an individual's locomotor adaptation capacity. Despite a large body of literature on split-belt adaptation in individuals of multiple ages and neuropathologies, surprisingly, split-belt adaptation has not been assessed in AD participants. We hypothesize that decreased capacity for split-belt adaptation may be an important contributing factor and a potential indicator of increased fall risk and cognitive decline in older individuals with MCI and AD. There is a need to understand how the split-belt adaptation task relates to cognitive deficits and walking function in individuals with a high risk of falls.

Herein, we utilized the split-belt adaptation task to compare the capacity for motor adaptation between a group of older adults with cognitive impairment (MCI, AD) and age-matched healthy controls. We also evaluated the hypothesis that locomotor adaptation capacity would be associated with cognitive function. To our knowledge, this is the first analysis of locomotor adaptation and its relationships with cognition in MCI and AD individuals.

## Materials and Methods

All study procedures were approved by the Emory Institutional Review Board, and all participants provided informed written consent.

### Subjects

All subjects were recruited from the Emory Alzheimer's Disease Research Center Registry. These subjects had undergone standard evaluations including measures that comprise the Uniform Data Set of the National Alzheimer's Coordinating Center. HOA subjects had received a diagnosis of normal cognition within 6 months before completing the study, while MCI and AD subjects received a diagnosis of MCI or AD, respectively, within 6 months before completing the study protocol. The MCI and AD subjects were grouped together as MCI/AD for data analysis. All subjects had no history of psychiatric (Axis I) disorders, alcohol/substance-related abuse, and neurologic conditions such as stroke or Parkinson's disease. Additionally, the subjects had no current significant alcohol use, were not taking hypoglycemic agents, no newly diagnosed neurologic conditions, and no orthopedic problems in the lower limbs or spine that limit walking.

### Lab Equipment

A 7-camera motion capture system (Vicon Inc., Colorado, USA) and an instrumented split-belt treadmill (Bertec Corporation, Ohio, USA) were used to collect marker and ground reaction force data during the walking assessment. Retro-reflective markers were attached to the subjects' upper back, pelvis, bilateral hip, knee, and ankle joints with adhesive skin tape, as detailed in our previous publications (21). The split-belt treadmill allows the two belt speeds to be operated independently, enabling different belt speeds for each leg. While walking on the treadmill, the subjects wore a safety harness without body weight support suspended from a roof-mounted support rail. The subjects had access to a front handrail during treadmill walking and were allowed to hold on to the handrail as needed during data-collection. When using the handrail, subjects were instructed to maintain a consistent handrail grip throughout the session.

### Walking Assessment

**Figure 1:**
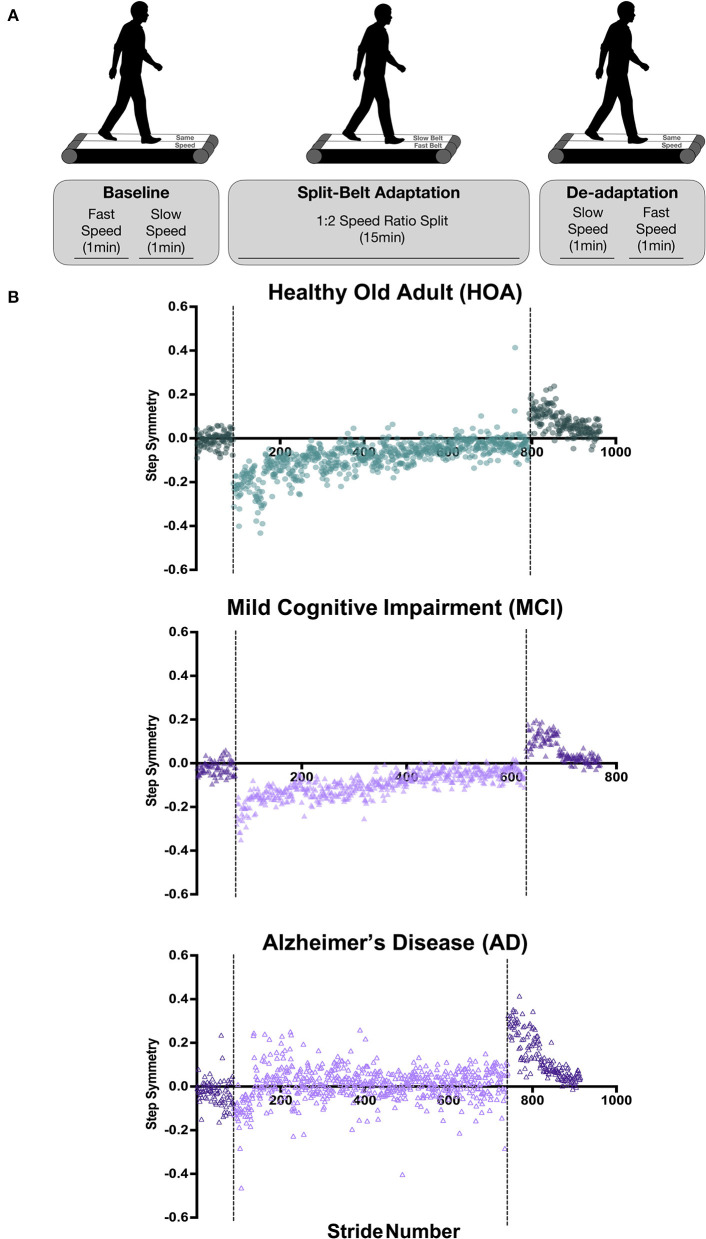
Split-belt walking adaptation protocol and individual participant step symmetry data. **(A)** During baseline (tied-belt) walking, participants walked at their self-selected “fast” speed for 1 min followed by 1-min walking at 50% of the self-selected speed, deemed the “slow” speed. During the split-belt adaptation period, the treadmill belt under the participant's dominant leg was set to the fast speed, whereas the non-dominant leg was set to slow speed for 15 min. The de-adaptation period involved 1-min walking with belts tied at the slow speed, followed by 1 min tied at the fast speed. **(B)** Step symmetry for individual participants throughout the duration of the experiment. Strides that occurred during tied belt walking are depicted as a darker color than the lighter-colored split-belt walking strides. Baseline walking is shown prior to the first dotted line, split-belt walking adaptation period is shown between the dotted lines, and de-adaptation walking trials can be found after the second dotted line. Note that the HOA participant data are shown in green circles, MCI participant data in purple filled triangles, AD participant in purple unfilled triangles.

The walking assessment consisted of three phases: a baseline phase in which the belts operated at the same speed (Pre-tied), a phase in which the belts operated at different speeds (Split-belt), and a final phase in which the belts operated at the same speed (Post-tied) (Figure 1). At the start of the session, the subject's self-selected walking speed was assessed by slowly increasing the treadmill belt speed to ascertain the subject's self-selected comfortable gait speed. This self-selected speed was designated as the “fast” speed and 50% of the self-selected speed was designated as the “slow” speed. Additionally, subjects were asked which leg was their dominant leg, by asking which leg they would use to kick a ball. Throughout the different phases of the split-belt walking session, the subjects were informed when the treadmill was going to start speeding up, slowing down, or going to be split. Subjects were instructed to look straight ahead and refrain from looking down at their feet to avoid any visual feedback regarding belt speeds.

### Pre-Tied Phase

After assessing the subject's self-selected speed, data were collected during the pre-tied phase, with the subject walking on the treadmill with belt speeds tied for 1 min at the fast speed, followed by 1 min at the slow speed.

### Split-Belt Phase

Following the pre-tied phase, the belt underneath the subject's dominant leg was increased to the fast speed, while the belt underneath the non-dominant leg remained at the slow speed. Thus, the treadmill belt speeds were split to a 2:1 speed ratio. This change in speed induced an initial asymmetry or limp in the subject's gait pattern. The subject continued to walk with this split-belt adaptation condition for 15 min. Gait data collected during this period were used to evaluate each individual's locomotor adaptation capacity by assessing the difference in inter-limb step symmetry that the split-belt induced, and the number of steps required to reach a plateau in step symmetry.

### Post-Tied Phase

After the conclusion of the split-belt phase, during the post-adaptation period, the belt moving at the fast speed was returned to the slow speed. The subject walked at this tied-belt slow speed for 2 min. Then, both belts increased to the fast speed, and the subject walked for an additional 2 min. After 2 min of fast walking, both belts slowed to a stop. Gait data from this phase were used to evaluate aftereffects or the locomotor system's ability to de-adapt following the split-belt adaptation.

### Cognitive Assessment

Following the treadmill assessment, the experimenters administered the Montreal Cognitive Assessment (MoCA) and the n-back subtests of the NIH EXAMINER (Executive Abilities: Measures and Instruments for Neurobehavioral Evaluation and Research).

### Data Processing

Marker data were labeled using Vicon Nexus software and then transferred to Visual 3D software (C-Motion, Inc., Maryland, USA) for further processing. Bilateral step lengths were calculated as the antero-posterior distance between the heel markers of the leading foot and the trailing foot at heel strike. Step length was defined with reference to the leading leg (i.e., ‘fast step length' corresponds to the step length when the foot on the fast belt is the leading foot). To compare the fast and slow steps, step length symmetry was calculated for each step as follows (22):


(1) $$\begin{array}{r} Step\;symmetry=\;\frac{\left(Fast\;step\;length\;-slow\;step\;length\right)}{\left(Fast\;step\;length\;+\;slow\;step\;length\right)}\; \end{array}$$

Using this formula, a step symmetry of zero would correspond to equal step lengths for both the fast and slow steps.

Step symmetry data for the split-belt and post-tied periods were normalized for each individual by subtracting with respect to the average of the last 5 steps of the pre-tied period. Therefore, a step symmetry equal to zero for each individual corresponds to that individual's baseline step symmetry.

Four periods were primarily used to assess the magnitude of adaptation and de-adaptation (19):

- Early adaptation: mean of first five steps of the split-belt period.
- Late adaptation: mean of last five steps of the split-belt period.
- Early aftereffects: mean of first five steps of the post-tied period.
- Late aftereffects: mean of last five steps of the post-tied period.

The early adaptation step symmetry is also referred to as the **magnitude of adaptation** since it is the initial magnitude of change induced at the beginning of the split-belt adaptation period.

The **rate of adaptation** was defined as the number of steps taken after the split-belt period begins for the subject to reach the adaptation plateau, defined as the average step symmetry of the last 30 steps of the split-belt period. A custom MatLab (The MathWorks, Inc., Massachusetts, USA) program was used to compare the average step symmetry of every five steps with the step symmetry in the plateau window, defined as the adaptation plateau ± the standard deviation of the step symmetry of the last 30 steps. The plateau was considered to be reached when five consecutive 5-step averages were within the plateau window. The rate of adaptation was then defined as the step number of the first of those five consecutive 5-step averages.

The **rate of de-adaptation** was calculated in the same manner, with the exception that the plateau was calculated as the mean of the last five steps (instead of the last 30 steps) because the de-adaptation period did not contain as many steps as the split-belt period.

### Statistical Analysis

The primary dependent variables for analysis were **step symmetry, magnitude of adaptation, and rate of adaptation**. A 2-way ANOVA was used to evaluate the effect of group (HOA, MCI/AD) and time (early adaptation, late adaptation, early aftereffects, late aftereffects) on step symmetry. A 1-way ANOVA was used to evaluate the effect of group (HOA, MCI/AD) on the magnitude of adaptation (signed values and not absolute values) and the rate of adaptation. *Post-hoc t*-tests were used for specific comparisons that showed differences after completing the ANOVAs. Secondary variables included MOCA and n-back scores. *T*-tests were performed to evaluate the difference in MOCA scores and n-back scores between the HOA and MCI/AD groups. Pearson correlations were computed to detect correlations between the primary (locomotor) and secondary (cognitive) variables. SPSS version 24 (IBM) was used for all statistical analyses. We also similarly included analysis on belt speeds to evaluate whether group differences in belt speeds influence adaptation. Alpha level was set as 0.05.

## Results

Participant demographics are listed in Table 1. A total of 15 subjects completed the study protocol: 8 healthy old adults (HOA; age: 69.6 ± 1.5 years), and 7 subjects in the MCI/AD group−5 older adults with mild cognitive impairment (MCI; age: 70.2 ± 7.3 years), and 2 older adults with Alzheimer's disease (AD; age: 63.0 ± 5.7 years).

**Table 1:** Participant demographics.

**Variables**		**Healthy Old Adults (HOA)**	**Mild Cognitive Impairment (MCI)**	**Alzheimer's Disease (AD)**
		***n* = 8**	***n* = 5**	***n* = 2**
Age (yr)		69.6 ± 1.5	70.2 ± 7.3	63.0 ± 5.7
Height (cm)		155.6 ± 5.7	151.9 ± 6.8	159.2 ± 8.9
Weight (kg)		59.9 ± 9.7	54.9 ± 7.8	52.1 ± 12.9
Education level (yr)		12.4 ± 2.7	11.0 ± 1.9	12.0 ± 1.0
Female: Male		5: 4	3: 2	1: 1
MOCA (score)		28.75 ± 1.58	21.5 ± 3.35	14.0 ± 7.07
Slow belt speed (mps)		0.41 ± 0.07	0.36 ± 0.08	0.40 ± 0
Fast belt speed (mps)		0.82 ± 0.14	0.72 ± 0.15	0.80 ± 0

### Belt Speeds

A two-way ANOVA found no significant difference in belt speeds (slow or fast) between HOA and MCI/AD groups (Tables 1, 2). A Pearson's correlation analysis also did not detect any correlations between belt speed and adaptation magnitude, adaptation rate, de-adaptation magnitude, or de-adaptation rate (Table 2).

**Table 2:** Statistical results.

**Analysis**		**Figure**	***p*-value**
Two-Way ANOVA	Belt speeds in HOA vs. MCI/AD	Table 1	slow belt: 0.909 fast belt: 0.550
Pearson's correlation analysis	Belt speed vs. adaptation magnitude		0.979
Pearson's correlation analysis	Belt speed vs. adaptation rate		0.950
Pearson's correlation analysis	Belt speed vs. de-adaptation magnitude		0.692
Pearson's correlation analysis	Belt speed vs. de-adaptation rate		0.144
One-Way ANOVA	Average baseline for HOA vs. MCI/AD	Figures 2A,B	0.532
One-way ANOVA	Magnitude of adaptation for HOA vs. MCI/AD		0.0098^*^
One-Way ANOVA	Rate of adaptation for HOA vs. MCI/AD		0.943
One-Way ANOVA	De-adaptation magnitude for HOA vs. MCI/AD		0.289
One-Way ANOVA	Rate of deadaptation for HOA and MCI/AD		0.140
Two-Way ANOVA	Group (HOA, MCI/AD) and time (early adaptation, late adaptation, early aftereffects, late aftereffects) on step symmetry	Figure 2A	Group: 0.009^*^ Time: <0.001^*^ Interaction: 0.324
Planned, pairwise comparison	Each time point (early adaptation, late adaptation, early aftereffects, late aftereffects) pooled across groups		all <0.003^*^
Planned, pairwise comparison	Early adaptation vs. late adaptation, early aftereffects and late aftereffects.		all <0.001^*^
Planned, pairwise comparison	Late adaptation vs. early aftereffects		<0.001^*^
Planned, pairwise comparison	Late adaptation vs. late aftereffects		0.001^*^
Planned, pairwise comparison	Early aftereffects vs. late aftereffects		0.002^*^
Planned, pairwise comparison	HOA vs. MCI/AD at early adaptation	Figure 2A	0.010^*^
Planned, pairwise comparison	HOA vs. MCI/AD at late adaptation		0.299
Planned, pairwise comparison	HOA vs. MCI/AD at early aftereffects		0.139
Planned, pairwise comparison	HOA vs. MCI/AD at late aftereffects		0.289
One-Way ANOVA	HOA vs. MCI/AD MOCA scores		<0.001^*^
One-Way ANOVA	HOA vs. MCI/AD n-back scores		0.005^*^
Pearson's correlation analysis	MOCA score vs. early adaptation magnitude	Figure 3A	*p* = 0.024, *R^2^* = 0.335
Pearson's correlation analysis	N-back score vs. early adaptation magnitude	Figure 3B	*p* = 0.012, *R^2^* = 0.398
Pearson's correlation analysis	MOCA score vs. early de-adaptation magnitude	Figure 3C	*p* = 0.028, *R^2^* = 0.319,
Pearson's correlation analysis	N-back score vs. early de-adaptation magnitude	Figure 3D	*p* = 0.008, *R^2^* = 0.428
Pearson's correlation analysis	MOCA score and adaptation plateau	Figure 3E	*p* = 0.087, *R^2^* = 0.209

* *p < 0.05*.

### Pre-Tied Step Symmetry

**Figure 2:**
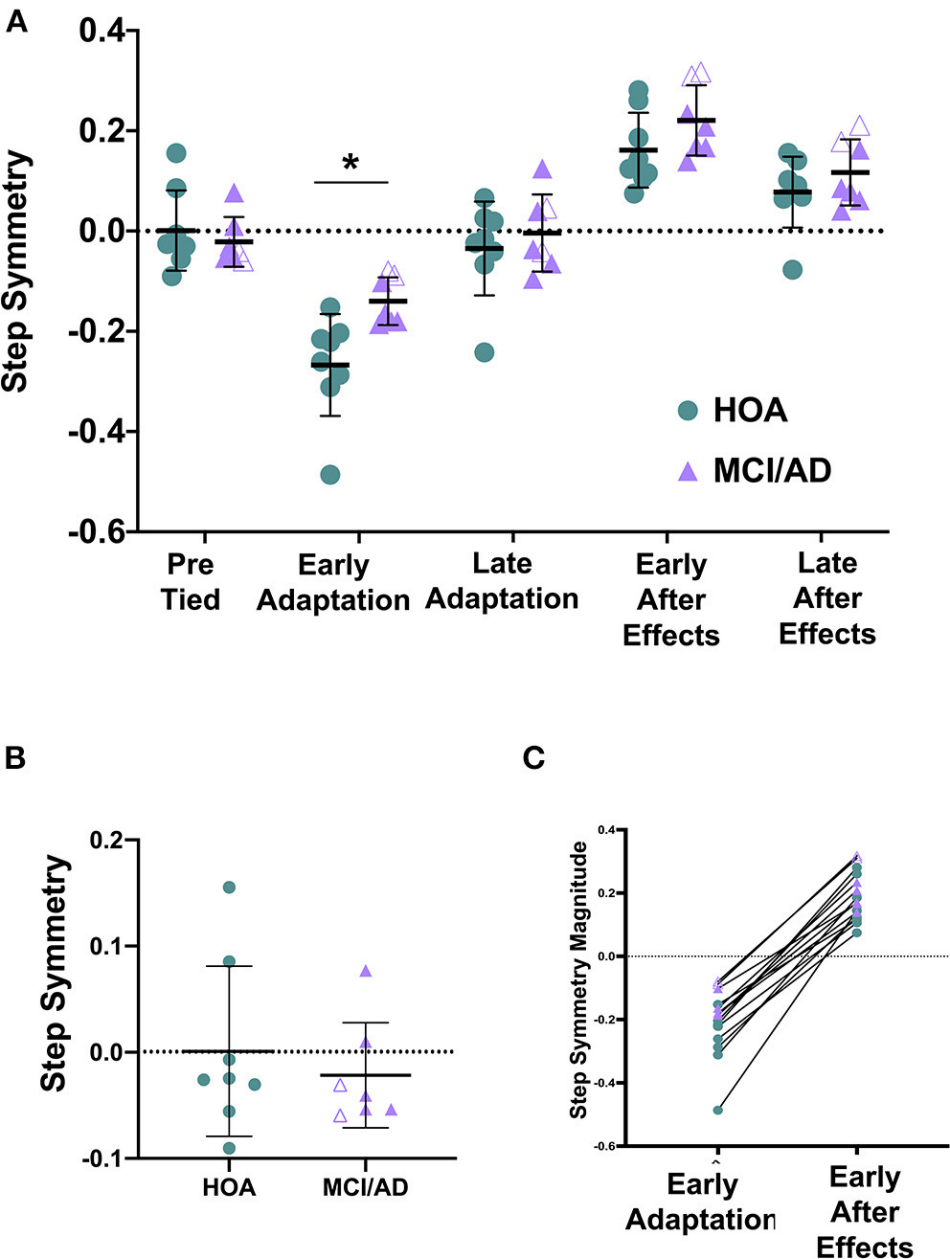
The magnitude of Early Adaptation is significantly reduced in MCI/AD compared to HOA. **(A)** Two-way ANOVA revealed a significant main effect of group (*p* = 0.009) and time (*p* < 0.0001) on step symmetry. Pairwise comparisons between groups revealed a significant difference between HOA and MCI/AD at early adaptation (*p = 0.0098). **(B)** Unpaired *t*-tests depicted no significant difference in step symmetry during baseline or tied-belt walking between HOA and MCI/AD (*p* = 0.532). **(C)** Comparison of step symmetry (magnitude) during early adaptation and early de-adaptation. Note that the HOA participant data are shown in green circles, MCI participant data in purple filled triangles, AD participant in purple unfilled triangles.

The average baseline or pre-tied step symmetry for HOA (0.001 ± 0.080) and MCI/AD (−0.022 ± 0.049) revealed no difference between groups (Figures 2A,B, Table 2).

### Magnitude and Rate of Adaptation and Aftereffects

The one-way ANOVA revealed a larger magnitude of adaptation for HOA (−0.267 ± 0.102) compared to MCI/AD (−0.140 ± 0.048) (Table 2). The ANOVA revealed no difference in the rate of adaptation for HOA (258.6 ± 171.6 steps) compared to MCI/AD (286.7 ± 138.0 steps) (Table 2).

The one-way ANOVA revealed no significant difference in magnitude of de-adaptation for HOA compared to MCI/AD (Table 2). Similarly, the ANOVA revealed no difference in the rate of de-adaptation for HOA compared to MCI/AD (Table 2).

### Comparison of Step Symmetry During Adaptation and Aftereffects

The 2-way ANOVA evaluating the effect of group (HOA, MCI/AD) and time (early adaptation, late adaptation, early aftereffects, late aftereffects) on step symmetry revealed a significant main effect of group and time. There was no interaction effect (Figure 2A, Table 2).

Planned, pairwise comparisons revealed a significant difference between each time point pooled across groups (Table 2). Planned, pairwise comparisons pooled across groups revealed a significant difference between early adaptation (−0.208 ± 0.102) vs. late adaptation (−0.011 ± 0.096), early aftereffects (0.189 ± 0.077), and late aftereffects (0.096 ± 0.069) (Table 2). Additionally, there were differences between late adaptation vs. early aftereffects, late adaptation vs. late aftereffects, and early aftereffects vs. late aftereffects (Table 2).

Planned, pairwise comparisons between groups revealed a significant difference between HOA and MCI/AD at early adaptation (Figure 2A, Table 2). There was no significant difference between HOA and MCI/AD at late adaptation (HOA = −0.036 ± 0.116, MCI/AD = 0.018 ± 0.064), early aftereffects (HOA = 0.161 ± 0.0.075, MCI/AD = 0.221 ± 0.070), or late aftereffects (HOA = 0.078 ± 0.071, MCI/AD = 0.117 ± 0.066) (Table 2).

### Cognitive Outcome Variables and Their Relationship With Adaptation

A significant difference was observed in the MOCA scores between HOA (28.8 ± 1.6) and MCI/AD (18.9 ± 5.2), as well as the n-back scores between HOA (0.84 ± 0.07) and MCI/AD (0.63 ± 0.16) (Table 2). For both MOCA and n-back tests, a higher score relates to better cognitive status.

**Figure 3:**
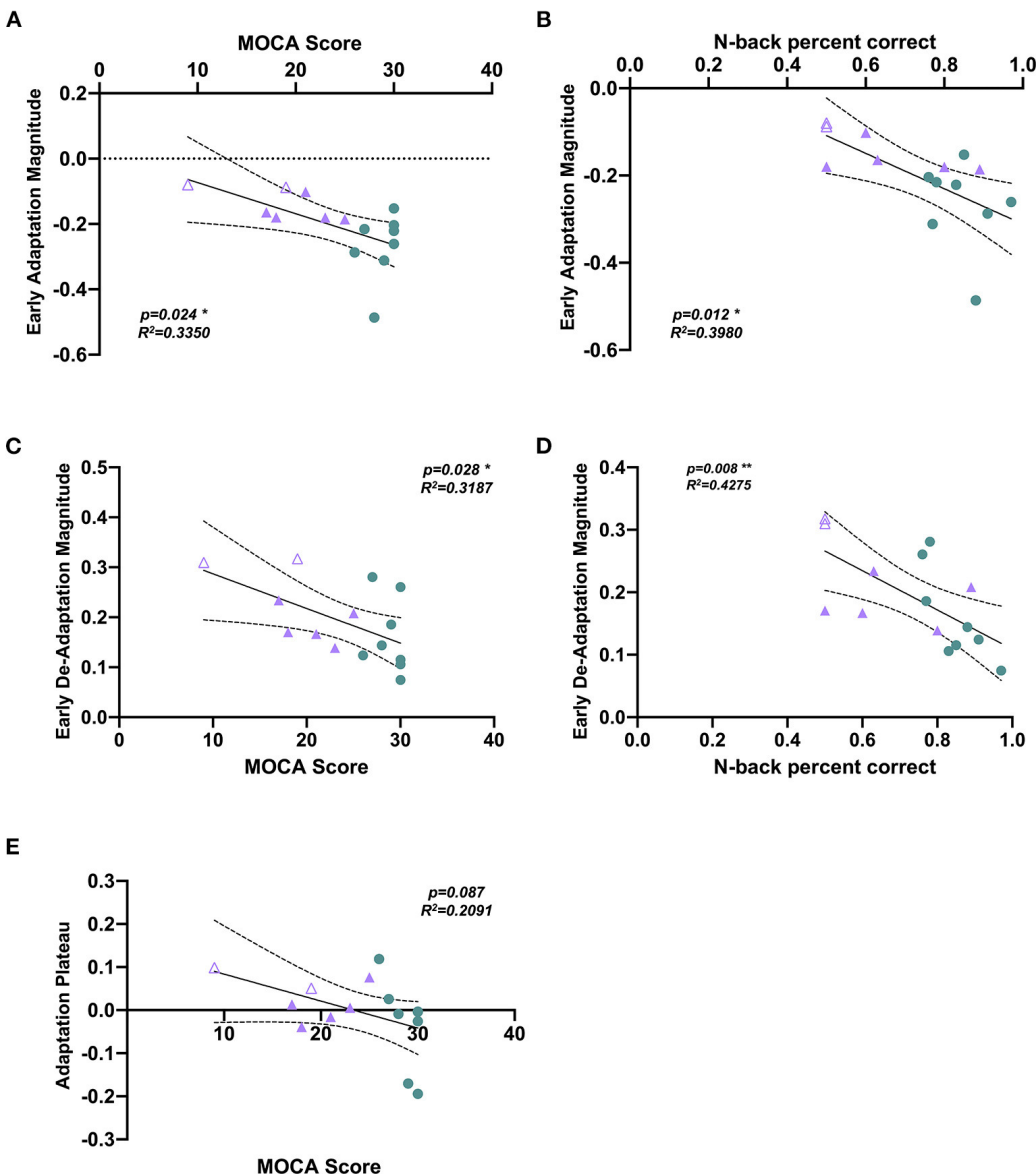
Scatterplots showing correlations between cognitive impairment measures and split-belt walking adaptation measures. A significant Pearson's correlation was observed between **(A)** MOCA score and early adaptation magnitude (*p* = 0.024, *R*^2^= 0.3350), with a higher MOCA score correlating to a greater magnitude of adaptation. **(B)** n-back correction rate and early adaptation magnitude (*p* = 0.012, *R*^2^ = 0.3980), with a higher n-back accuracy correlating to a greater magnitude of adaptation. **(C)** MOCA score and early de-adaptation magnitude (*p* = 0.024, *R*^2^ = 0.3350), with a higher MOCA score correlating to a lesser magnitude of de-adaptation. **(D)** n-back correction rate and early de-adaptation magnitude (*p* = 0.008, *R*^2^ = 0.4275), with a higher n-back accuracy correlating to a lesser magnitude of de-adaptation. **(E)** Correlation analysis between MOCA score and adaptation plateau (*p* = 0.087, *R*^2^ = 0.2091). Note that the HOA participant data are shown in green circles, MCI participant data in purple filled triangles, AD participant in purple unfilled triangles. *p < 0.05, **p < 0.005.

Pearson correlation analyses revealed a significant relationship between MOCA score and early adaptation magnitude, with a higher MOCA score (better cognitive status) correlating to a greater magnitude of adaptation (Figure 3A, Table 2). Similarly, Pearson correlation analyses revealed a significant correlation between n-back score and early adaptation magnitude, with a higher n-back score (better cognitive status) correlating to a greater magnitude of adaptation (Figure 3B, Table 2). Pearson correlation analyses also revealed a significant correlation between MOCA score and early de-adaptation magnitude, with a higher MOCA score (better cognitive status) correlating to a lesser magnitude of de-adaptation (Figure 3C, Table 2). Similarly, Pearson correlation analyses revealed a significant correlation between n-back score and early de-adaptation magnitude, with a higher n-back score (better cognitive status) correlating to a lesser magnitude of de-adaptation (Figure 3D, Table 2). A trend toward a significant correlation between MOCA score and adaptation plateau was found, with a higher MOCA score (better cognitive status) correlating with a more symmetrical step symmetry plateau during the adaptation period (Figure 3E, Table 2).

## Discussion

We found a significant difference in split-belt locomotor adaptation between healthy older adults (HOA) and older individuals with mild cognitive impairments and Alzheimer's disease (MCI/AD). Individuals with MCI/AD showed a significantly reduced magnitude of locomotor adaptation (i.e., magnitude of step symmetry during the early adaptation phase of split-belt walking). We found no between-group differences in baseline (i.e., pre-tied) step length symmetry or in the magnitudes and rate of de-adaptation. While our small sample preliminary study did not reveal differences in the rate of adaptation between HOA and MCI/AD, we observed much higher inter-individual variability in the time course and patterns of adaptation in individuals with MCI/AD. Furthermore, our correlation analyses revealed that individuals who showed smaller magnitudes of adaptation also demonstrated greater cognitive impairment (i.e., poorer MOCA and n-back scores). Interestingly, although there were no between-group differences during the de-adaptation phase of split-belt walking, we also found significant correlations between the magnitude of de-adaptation and cognitive impairment. Our study takes the first step toward our long-term goal of elucidating mechanisms underlying locomotor adaptation dysfunction and fall risk in older individuals with cognitive impairment.

Revealing a significant effect of group (HOA, MCI/AD), our results depicted a significantly reduced adaptation ability in cognitively impaired participants during early adaptation compared to healthy age-matched controls. We did not find significant differences during de-adaptation. Additionally, based on lack of significant differences in and correlations with belt speed, we infer that belt speed was not a major contributing factor for our observed effects on adaptation. Step-symmetry differences observed in HOA during early adaptation are somewhat consistent with previous literature on changes in gait with aging (22). Bruijn et al. (22) showed that HOA adapt less and more slowly, showing fewer aftereffects compared to young adults. Under the premise that cognition tends to decline with age, our study agrees somewhat with Bruijn et al. (22) in that our more cognitively impaired group (i.e., MCI/AD) showed a lesser magnitude of adaptation. Bruijn et al. (22) also noted a small sample size as a limitation of their work. In another previous study, Wolpea et al. (23) found a smaller magnitude of adaptation in HOA than young adults during visuomotor rotation learning tasks. The observed changes in locomotor adaptation with cognitive decline are also supported by longitudinal studies showing a decline in gait speed with AD progression, exacerbated by the performance of dual-task paradigms (15, 17). Studies observing changes in gait with cognitive decline during natural aging suggest that HOA demonstrate alterations in the locomotor system and adaptation strategies to maintain stability (24–26). Further cognitive decline, in the case of MCI/AD, may induce additional changes in the locomotor system and adaptation, which merit deeper investigation in future studies.

Walking in the real-world environment places high demands on the interplay between cognitive (i.e., executive function, working memory, and attention) and motor functions to adapt walking to rapidly evolving situations, terrains, and weather conditions. A well-functioning ability to sustain, shift, and divide attention between environmental and body function factors is essential for safe ambulation in everyday life. Unfortunately, cognitive dysfunction, the hallmark of MCI and AD, directly impacts the cognitive-motor neural resources available to carry out such activities of daily living (1, 2). Therefore, in addition to the hallmark cognitive features of MCI and AD, loss of independent mobility induced by balance and gait dysfunction is becoming increasingly recognized (27–29). This is consistent with our findings of significant correlations between the magnitude of adaptation and level of cognitive impairment. Importantly, our results show that individuals who have cognitive impairments may also demonstrate impairments in locomotor adaptation.

Split-belt walking, a unique adaptation task that induces complex asymmetries in the spatial and temporal coordination of walking patterns, has been used to investigate locomotor adaptation in various populations (18, 30–32). The split-belt method provides an advantage because it involves a standardized, robust, and well-studied locomotor task with potential implications for walking function, community participation, as well as fall prevention. Previously, split-belt has provided a robust measure of motor adaptation in children (33), young adults (34), elderly individuals (9, 22, 35, 36), stroke survivors (19, 20), people with Parkinson's disease (37, 38), and individuals with hemispherectomy (39). Furthermore, Malone and Bastian (31) showed a reduction in the rate of split-belt adaptation when able-bodied participants were distracted by a cognitive task during split-belt walking (31). Although we did not find a reduced rate of adaptation in MCI/AD, our finding of reduced adaptation magnitude in MCI/AD may suggest that cognitive impairments, somewhat similar to cognitive distraction, adversely affect the locomotor adaptation processes. These research questions need more in-depth study because to maintain stability and prevent falls during locomotion, human gait must be readily adapted in response to changes in internal and external environments. Similarly, and as noted, deficits in dual-tasking abilities have been shown to be related to increased gait variability and greater risk of falls (40). Individuals with MCI and AD, especially those with notable deficits in executive function, have difficulty with cognitive-motor dual-tasking, which may contribute to their fall risk (14, 16, 28, 40). Future studies could evaluate the effect of cognitive-motor dual-tasks during split-belt walking in people with MCI and AD.

Despite finding a significant difference in magnitude of adaptation, we observed no significant difference between HOA and MCI/AD for the magnitude of de-adaptation nor the rates of adaptation and de-adaptation. The lack of significant difference for the magnitude of de-adaptation may be due to a small sample size. Due to the small sample, MCI and AD were grouped together to represent cognitively impaired individuals; however, our individual subject data suggested that AD participants showed greater average de-adaptation magnitude compared to those with MCI, both of which were greater than HOA. Both MOCA and n-back scores showed significant relationships with the magnitude of adaptation, suggesting that individuals with greater cognitive impairment also demonstrate a reduced capacity to adapt their walking in response to the split-belt perturbation. These relationships suggest that cognitive status may be an important contributor to walking function and the risk of falls in older individuals with cognitive impairments. Furthermore, given the absence of between-group differences in de-adaptation, we were surprised to find significant associations between de-adaptation magnitude and cognition, such that individuals who showed a larger magnitude of de-adaptation also showed greater cognitive impairment. Notably, we found considerable inter-individual variability in the time course of adaptation and de-adaptation in people with greater cognitive impairment in our cohort. Additionally, individual participant data revealed several examples wherein a small magnitude of early adaptation was accompanied by a relatively large magnitude of de-adaptation (Figure 2c). The mechanisms underlying the somewhat disparate effects of MCI/AD on adaptation vs. de-adaptation processes merit further investigation in larger sample studies. Potentially, impairments in higher-order executive functions contribute to greater stride-to-stride variability during walking and adaptation, as well as a variable time course of response to the split-belt task. While correlation does not prove causation, future studies should probe potential factors causing locomotor adaptation deficits by implementing walking training comprising multiple sessions of split-belt walking, to evaluate whether improvements in locomotor adaptation are accompanied by improved cognitive function in people with MCI and AD.

Our findings have implications and provide a foundation for future inquiry aimed at understanding locomotor dysfunction in MCI and AD. However, this study is not without limitations. The most notable limitation of our study is the small sample size. Despite having a small sample size, our correlation results and differences between MCI and AD participants warrant further larger-sample investigations. With a larger sample size, sex differences in locomotion and adaptation could also be analyzed. Previous literature proposes that individuals may cope with gait disturbances *via* a “risky” adaptation (e.g., increased gait speed), as seen in dementia patients, or a “secure” adaptation (e.g., slowed gait speed and shortened stride length), common in HOA (17). Previous literature shows that repeated exposure to the split-belt adaptation task may improve locomotion in stroke survivors (9, 19, 41, 42). Despite these promising previous results, it is unknown if multiple sessions of split-belt adaptation could be successfully applied as a potential exercise-based treatment for enhancing walking function in MCI and/or AD, necessitating further study.

## Conclusions

Understandably, to date, the neural underpinnings and the related cognitive outcomes in MCI and AD are the primary focus of evaluation, treatment, and research aimed at lessening the disease progression and burden. However, considering the known importance of locomotion in maintaining the quality of life and the benefits of non-pharmacological exercise-based interventions for enhancing physical and cognitive function, our study aimed to understand the effects of MCI and AD on locomotor adaptation using a robust, standardized split-belt walking task. Our results showed a significant reduction in locomotor adaptation in MCI/AD compared to HOA and significant relationships between locomotor adaptation and cognitive function impairments. Future research is needed to better understand neuromechanical factors contributing to gait dysfunction in people with MCI and AD, the relationships between locomotor and cognitive impairments, and their association with disability, falls, and quality of life in individuals with MCI and AD.

## Data Availability Statement

The raw data supporting the conclusions of this article will be made available by the authors, without undue reservation.

## Ethics Statement

The studies involving human participants were reviewed and approved by Emory University Institutional Review Board. The patients/participants provided their written informed consent to participate in this study.

## Author Contributions

TK and JN conceived and designed research. TK, JN, and SE performed experiments and analyzed data. TP, TK, JN, and SE interpreted results of experiments. TP, TK, and SE prepared figures. TP, TK, and JN drafted, prepared, edited, revised, and edited the final versions of the manuscript. All authors approved the final version of manuscript.

## Funding

This work was supported by NIH NICHD Grants (K01 HD079584 and R01 HD09597) to TK, NIH NICHD Grant (K12HD055931) to JN, and NIH NINDS (T32NS096050) to TP. This work was also funded by a pilot project grant awarded to TK and JN from the Emory Alzheimer's Disease Research Center (ADRC).

## Conflict of Interest

The authors declare that the research was conducted in the absence of any commercial or financial relationships that could be construed as a potential conflict of interest.

## Publisher's Note

All claims expressed in this article are solely those of the authors and do not necessarily represent those of their affiliated organizations, or those of the publisher, the editors and the reviewers. Any product that may be evaluated in this article, or claim that may be made by its manufacturer, is not guaranteed or endorsed by the publisher.
